# About Illinois State Laws

**Published:** 1898-06

**Authors:** 


### ABOUT ILLINOIS STATE LAWS.

Diseases of horses and cattle only are provided for in State sanitary work. State Veterinarian Lovejoy strongly urges the extension of the laws to cover sheep, swine, and poultry.

Mallein has been extensively used during the past year in efforts to control glanders, by endeavoring to eliminate the obscure cases.

Chicago furnishes the largest number of glandered horses of the cities, while the southern section of the State is very prolific in these cases.

Of cattle inspected and condemned, about 9 per cent. seem to be affected with tuberculosis.

Lack of sufficient appropriation hampers the work in the "Prairie State." A conservative estimate places the loss from tuberculosis at about $90,000 per year. Surely a very unnecessary burden upon those engaged in the livestock industry.

A law is badly needed prohibiting the importation of cattle into the dairy and breeding districts without subjecting them to the tuberculin-test.

An appropriation of $25,000 a year, well expended, would soon make this a disease little known in Illinois. Members of the State Legislature should be made to look this question fairly in the face.

A more liberal education of the stockgrowers upon this question is very essential, and in these days when valuable literature on the subject is so abundant one would expect much progress. Every veterinarian should do some missionary work in this direction.

More of the cities should inaugurate the plan of demanding a certificate showing freedom from tuberculosis of the cattle supplying milk to these centres. This is an important line of work for every veterinarian.

Chicago should lead, not follow, this plan now adopted in a number of cities. A city so densely populated cannot afford to be without this safeguard for her people. Strengthen Commissioner of Health Reynolds' hands, and this will soon follow. The Chicago Veterinary Society can do much in this direction.

A law is wanted throughout the entire State compelling the removal of all milk-tanks and houses a proper distance from the cow-sheds and barns.

With so many assistant State veterinarians a police system of the State could be readily maintained, and thousands of dollars saved to her people at an expenditure of hundreds.

